# The oscillation of intracellular Ca^2+^ influx associated with the circadian expression of *Piezo1* and *TRPV4* in the bladder urothelium

**DOI:** 10.1038/s41598-018-23115-w

**Published:** 2018-04-09

**Authors:** Tatsuya Ihara, Takahiko Mitsui, Yuki Nakamura, Mie Kanda, Sachiko Tsuchiya, Satoru Kira, Hiroshi Nakagomi, Norifumi Sawada, Manabu Kamiyama, Yuri Hirayama, Eiji Shigetomi, Youichi Shinozaki, Mitsuharu Yoshiyama, Atsuhito Nakao, Masayuki Takeda, Schuichi Koizumi

**Affiliations:** 1 Department of Urology, Interdisciplinary Graduate School of Medicine, University of Yamanashi, Chuo, Yamanashi, Japan; 2 Department of Immunology, Interdisciplinary Graduate School of Medicine, University of Yamanashi, Chuo, Yamanashi, Japan; 3 Department of Neuropharmacology, Interdisciplinary Graduate School of Medicine, University of Yamanashi, Chuo, Yamanashi, Japan

## Abstract

We previously showed that bladder functions are controlled by clock genes with circadian rhythm. The sensation of bladder fullness (SBF) is sensed by mechano-sensor such as Piezo1 and TRPV4 in the mouse bladder urothelium. However, functional circadian rhythms of such mechano-sensors remain unknown. To investigate functional circadian changes of these mechano-sensors, we measured circadian changes in stretch-evoked intracellular Ca^2+^ influx ([Ca^2+^]_*i*_) using mouse primary cultured urothelial cells (MPCUCs). Using Ca^2+^ imaging, stretch-evoked [Ca^2+^]_*i*_ was quantified every 4 h in MPCUCs derived from wild-type (WT) and *Clock*^*Δ19/Δ19*^ mice, which showed a nocturia phenotype. Furthermore, a Piezo1 inhibitor GsMTx4 and a TRPV4 inhibitor Ruthenium Red were applied and stretch-evoked [Ca^2+^]_*i*_ in MPCUCs was measured to investigate their contribution to SBF. Stretch-evoked [Ca^2+^]_*i*_ showed a circadian rhythm in the WT mice. In contrast, *Clock*^*Δ19/Δ19*^ mice showed disrupted circadian rhythm. The administration of both GsMTx4 and Ruthenium Red eliminated the circadian rhythm of stretch-evoked [Ca^2+^]_*i*_ in WT mice. We conclude that SBF may have a circadian rhythm, which is created by functional circadian changes of Piezo1 and TRPV4 being controlled by clock genes to be active during wakefulness and inactive during sleep. Abnormalities of clock genes disrupt SBF, and induce nocturia.

## Introduction

Clock genes exist in most cells and organs, where they regulate the circadian rhythm. There are more than ten types of clock genes, whose products form complicated transcriptional feedback loops controlled by the master clock genes in the suprachiasmatic nucleus^1^. It has been reported that lower urinary tract functions also involve a circadian rhythm and *Clock* mutant (*Clock*^*Δ19/Δ19*^) mice urinated frequently in the sleep phase^2–4^. These reports suggested that malfunction of clock genes contributed to nocturia.

The urothelium can sense bladder extension and respond to stimuli with neurotransmission, partly controlled by adenosine triphosphate (ATP)^5,6^. Bladder fullness is sensed by mechano-sensors, such as transient receptor potential cation channel subfamily V member 4 (TRPV4) and Piezo Type Mechanosensitive Ion Channel Component 1 (Piezo1) via intracellular Ca2+ influx ([Ca^2+^]_*i*_)^7,8^, which introduces ATP release from the urothelium. In a previous study, we revealed that clock genes function to create circadian expressions of mechano-sensor genes such as *Piezo1* and *TRPV4* in the mouse bladder urothelium both *ex vivo and in vitro*^9,10^. Although the etiology of nocturia is still elusive, we hypothesized that abnormalities of clock genes elicit bladder hypersensitivity in the sleeping time due to the disruption of circadian rhythms of Piezo1 and TRPV4, which might be one of the causes of nocturia.

In this report, we checked whether the circadian rhythm of clock genes in the urothelium regulates bladder fullness through the circadian rhythm of [Ca^2+^]_*i*_ in the mouse primary cultured urothelial cells (MPCUCs).

## Results

### The circadian rhythm of [Ca^2+^]_*i*_ in MPCUCs

We examined the time-dependent changes in stretch-evoked [Ca^2+^]_*i*_ in wild-type (WT) and *Clock*^Δ19/Δ19^ mice.

**Figure 1:**
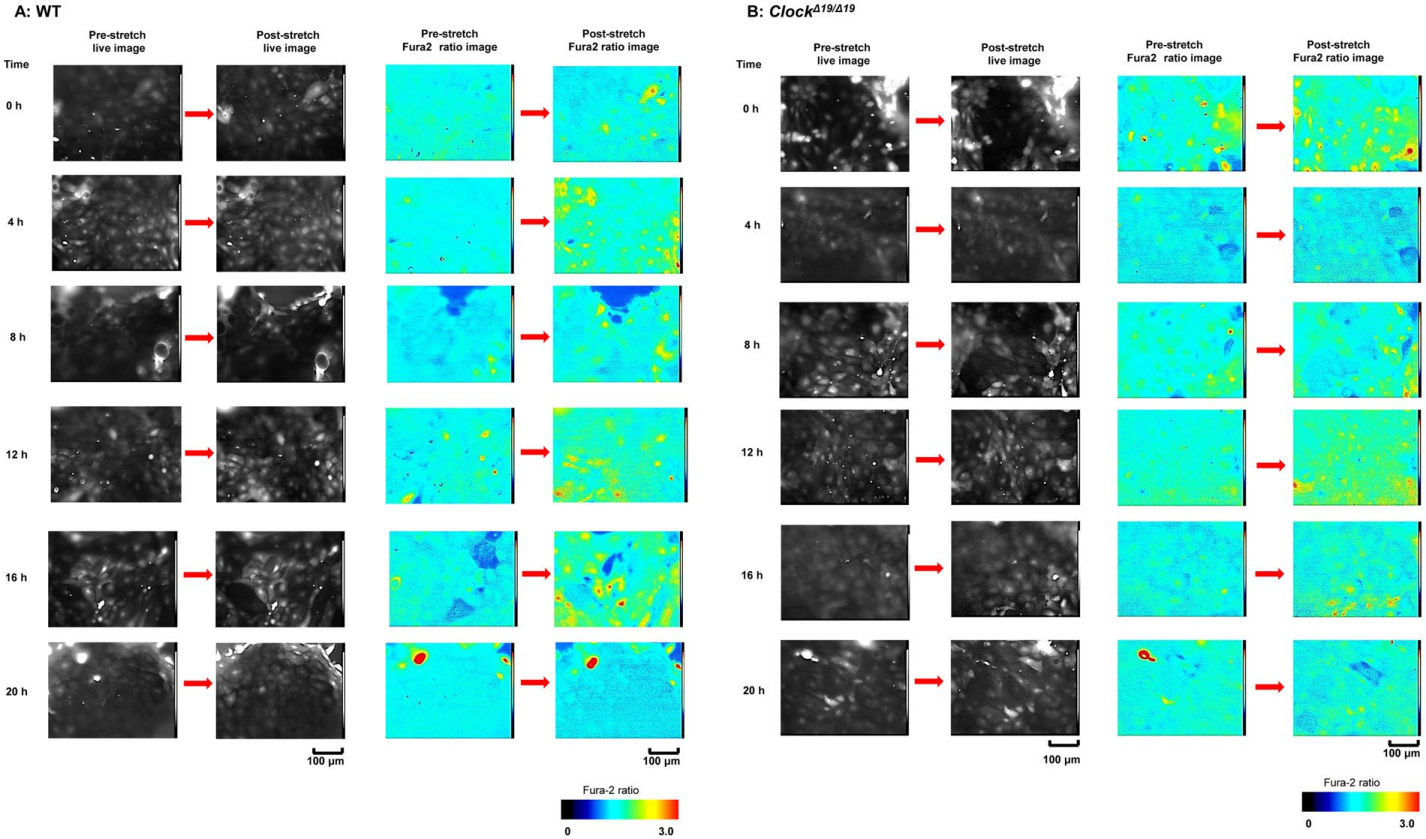
The live images and Fura2 ratio images at pre- and post-stretch. (**A**) Wild-type (WT) mice: The representative live images (left panels) and Fura2 ratio images (right panels) of mouse primary cultured urothelial cells (MPCUCs) in pre- and post-stretch from 0 to 20 h. (**B**) *Clock*^*Δ19/Δ19*^ mice: The representative live images (left panels) and Fura2 ratio images (right panels) of MPCUCs in pre- and post-stretch from 0 to 20 h. Time 0 means 12 hours after horse serum shock (HSS). All images were taken from cells seeded on the 1-mm slit area in the stretch chamber. The stretch speed was fixed at 100 μm/s, and the distance was 100 μm. Cells were extended transversely in both cell types. Scale bar: 100 μm.

**Figure 2:**
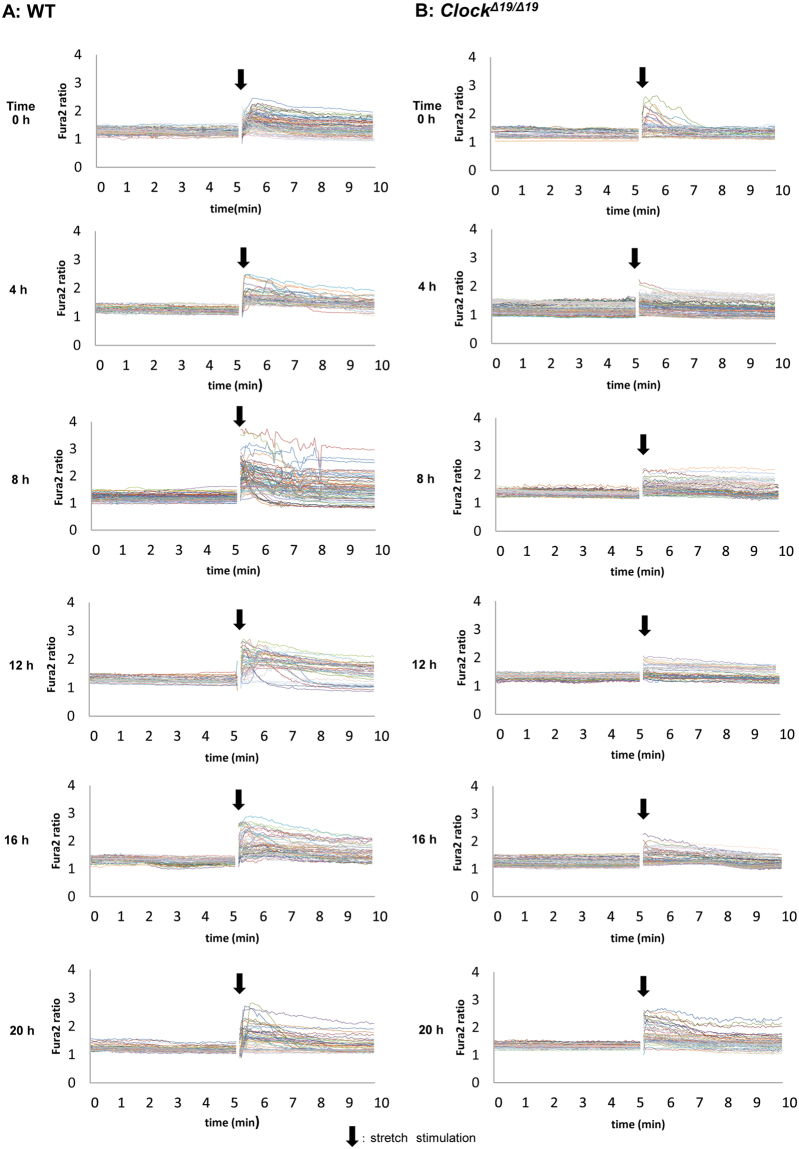
The traces of Fura2 ratio changes at pre- and post-stretch. A, Wild-type (WT) mice (left panels): The traces of Fura2 ratio changes in all analyzed cells from 0 to 20 h. B, *Clock*^*Δ19/Δ19*^ mice (right panels): The traces of Fura2 ratio changes in all analyzed cells from 0 to 20 h. The number of analyzed cells is 83, 52, 92, 41, 53, and 57 in the WT mice, and 46, 100, 75, 47, 76, and 58 in *Clock*^*Δ19/Δ19*^ mice from 0 to 20 h. Black arrows denote the onset of the stretch. Time 0 means 12 hours after horse serum shock (HSS). All images were taken from cells seeded on the 1 mm slit area in the stretch chamber. The stretch speed was fixed at 100 μm/s, and the distance was 100 μm. Cells were extended transversely in both cell types. Scale bar: 100 μm.

In both groups, the cell in the post-stretch conditions after Fura2 loading were different compared with cells in the pre-stretch conditions. A few cells in the field of view were observed to be removed from the silicon chamber after stretch stimulation, and the Fura2 ratios showed large changes around these removed cells (Fig. 1A,B). In WT mice, the largest increase in the Fura2 ratio occurred around 12 h at post-stretch (Fig. 1A, right panels) and this was confirmed by the traces of the Fura2 ratio changes shown in Fig. 2A. However, in the *Clock*^*Δ19/Δ19*^ mice, the time-dependent changes at post-stretch were lower or absent from 0 h to 20 h compared with WT mice (Fig. 1B, right panels). In the traces of the Fura2 ratio changes, the tendency of no change was observed and the peak values were almost constant from 0 h to 20 h, at around 2.5 (Fig. 2B). There was also a difference in the decreasing pattern after stretch stimulation. The increasing Fura2 ratio returned to the baseline in some cells, and other cells maintained higher level after stretch stimulation during measurement for 5 min (Fig. 2).

**Figure 3:**
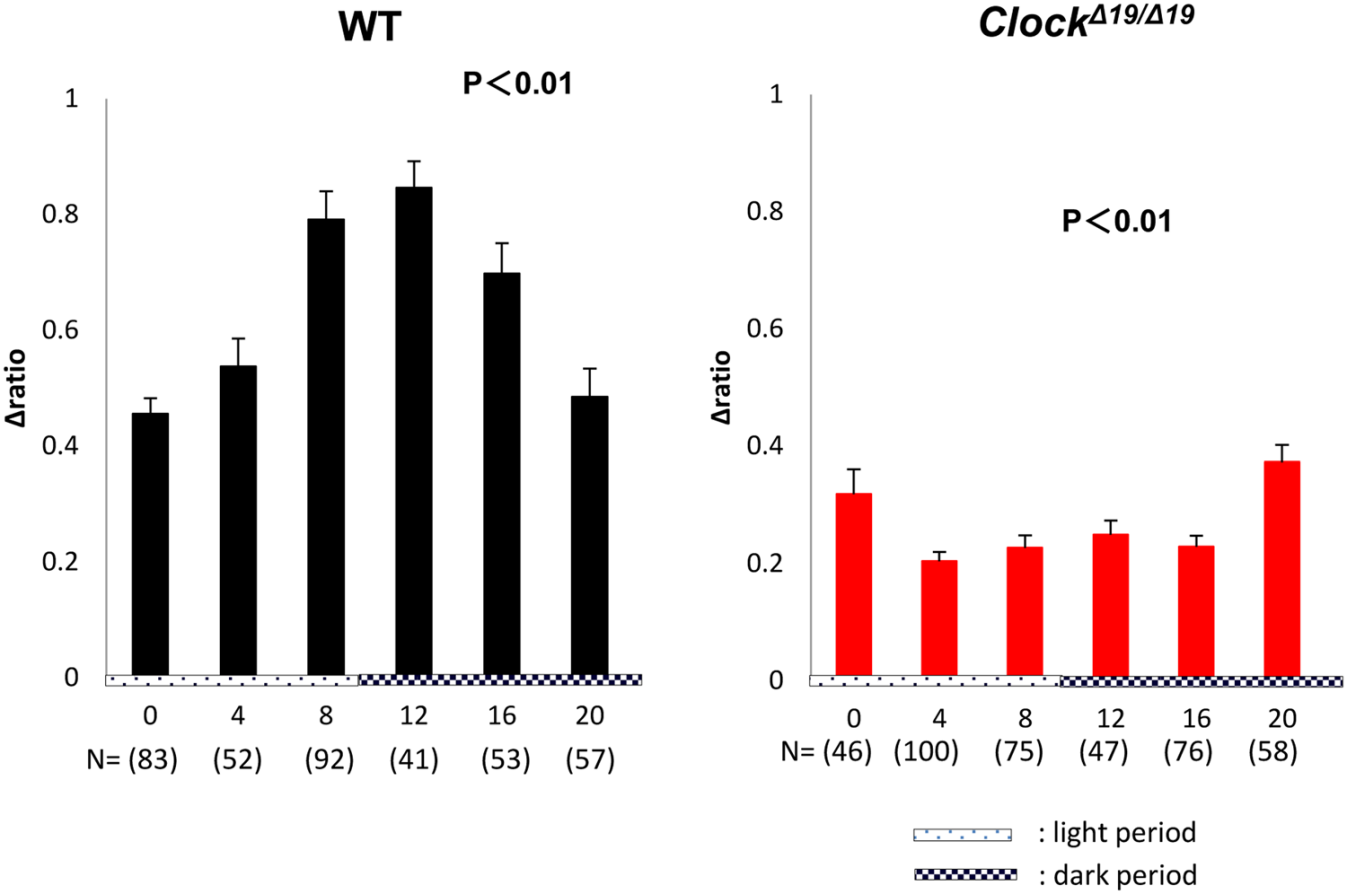
Time-dependent changes in the average peak of intracellular Ca^2+^ influx ([Ca^2+^]_*i*_) after stretch stimulation. The time-dependent changes in the average peak of [Ca^2+^]_*i*_ at post-stretch: Wild-type (WT) mice (black) and *Clock*^*Δ19/Δ19*^ mice (red). The number of analyzed cells is 83, 52, 92, 41, 53, and 57 in the WT mice, and N = 46, 100, 75, 47, 76, and 58 in *Clock*^*Δ19/Δ19*^ mice from 0 to 20 h. Data are presented as the means ± standard error (SE). The numbers of analyzed cells are indicated in parenthesis below the chart. The abscissa of the each graph is the time axis. 0 h means 12 hours after horse serum shock (HSS). Statistical analyses were done using a one-way analysis of variance (ANOVA) to compare differences among the time points in each group.

The time-dependent change in the average stretch-evoked [Ca^2+^]_*i*_ from 0 h to 20 h showed a circadian rhythm in the WT mice. The peak time was observed at 12 h, which corresponded to the beginning of the active phase, and the nadir was at 0 h, which corresponded to the beginning of the sleep phase (Fig. 3, left). *Clock*^*Δ19/Δ19*^ mice also showed significant time-dependent changes in the average stretch-evoked [Ca^2+^]_*i*_ (Fig. 3, right). However, this pattern was not the circadian rhythm observed in WT mice.

### The effect of extracellular Ca^2+^ and inhibition of mechano-sensors on the circadian rhythm of [Ca^2+^]_*i*_ in MPCUCs

**Figure 4:**
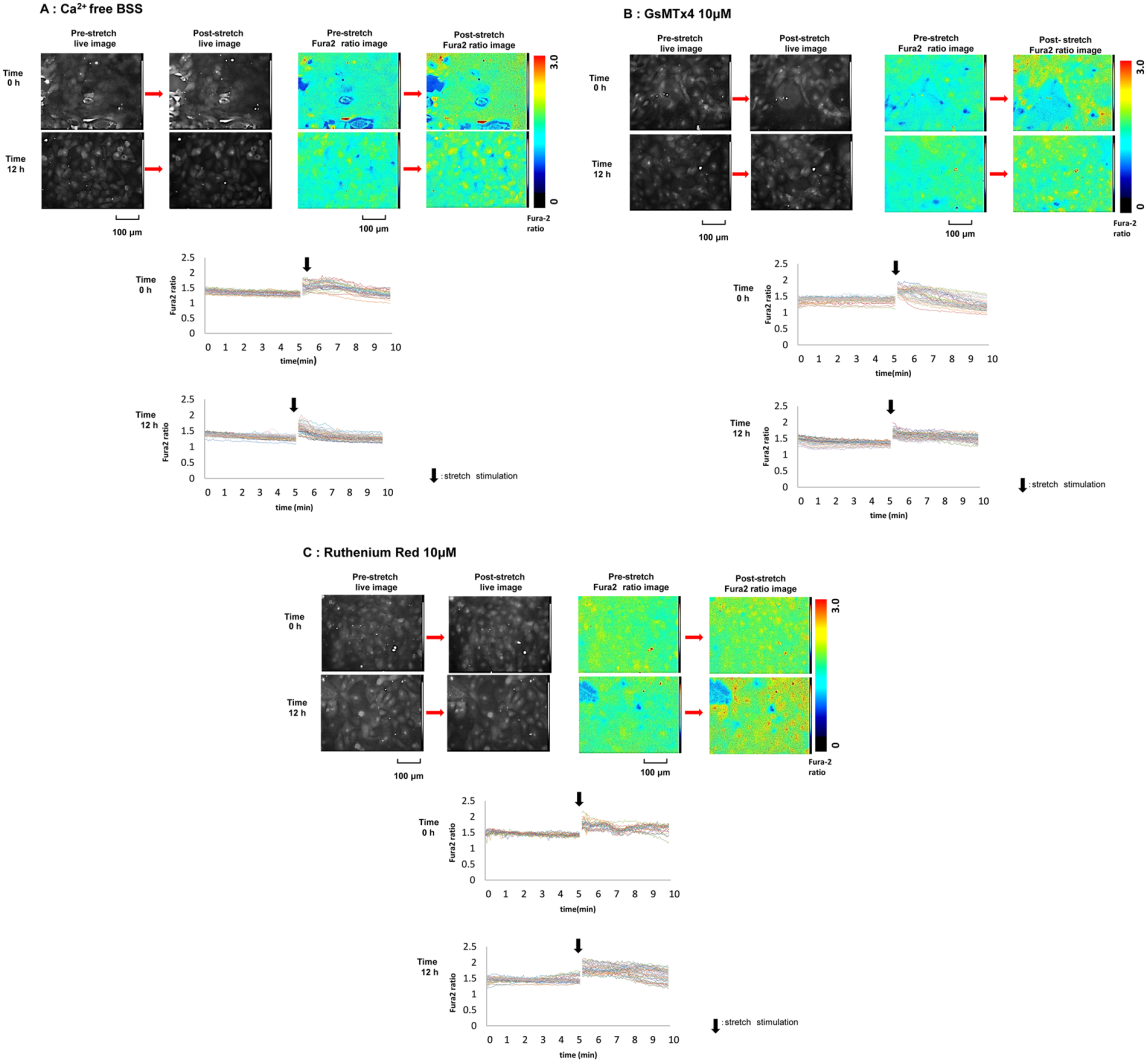
Images of Fura2 ratio at pre- and post-stretch under different conditions. The representative live images (upper left panels) and Fura2 ratio images (upper right panels) of primary cultured urothelial cells at pre- and post-stretch at difference time point, 0 and 12 h, and the traces of Fura2 ratio changes in all of analyzed cells (lower panels). (**A**) Ca^2+^ free balanced salt solution (BSS). (**B**) GsMTx4 10 μM treated conditions. (**C**) Ruthenium Red 10 μM treated conditions. Time 0 means 12 hours after horse serum shock (HSS). Black arrows denote the onset of the stretch. The stretch speed was fixed at 100 μm/s, and the distance was 100 μm. Cells were extended transversely in both cell types. Scale bar: 100 μm.

**Figure 5:**
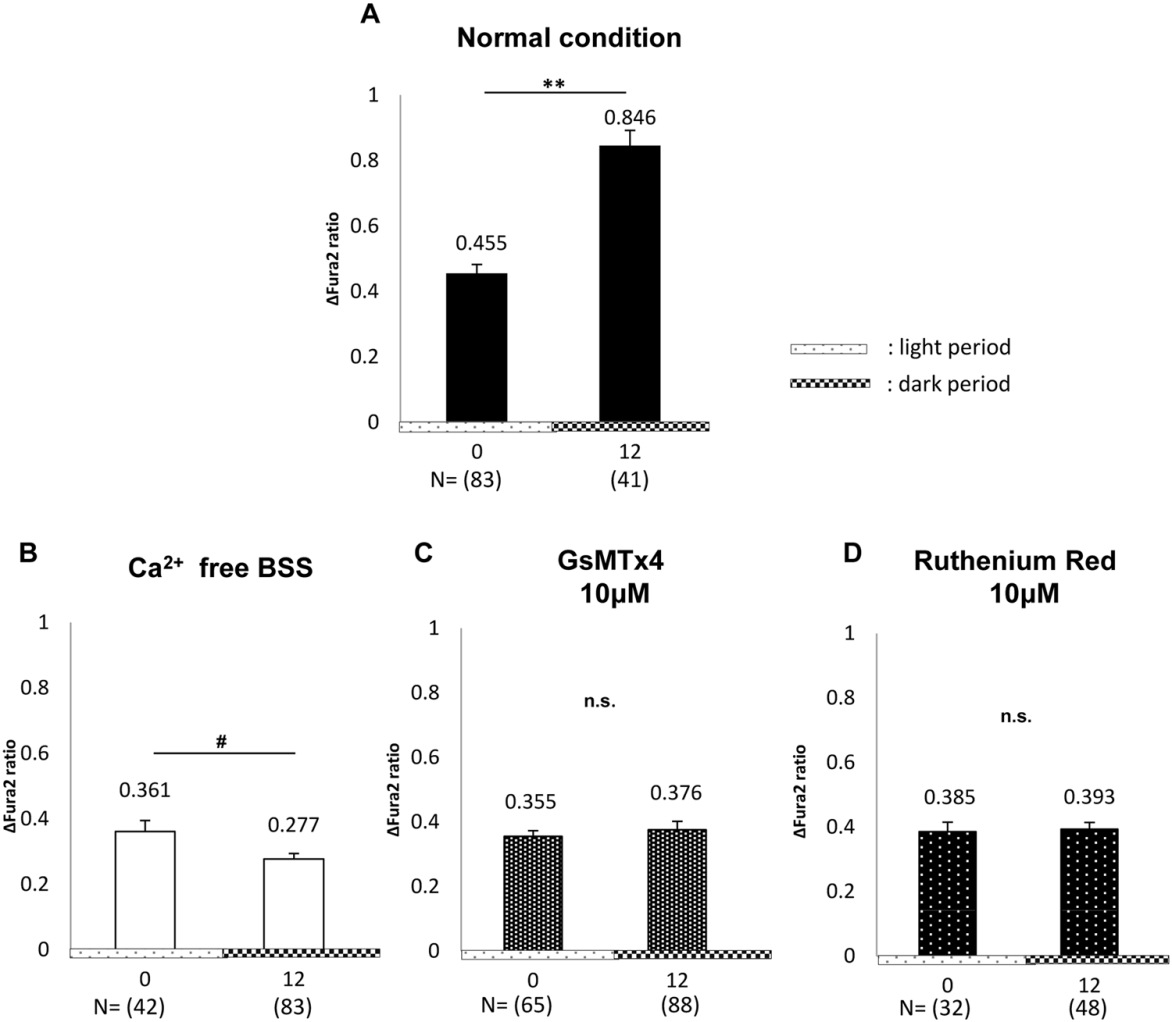
Effect of extracellular Ca^2+^, GsMTx4, and Ruthenium Red (RR) on intracellular Ca^2+^ influx ([Ca^2+^]_*i*_) after stretch stimulation. The changes of average peak of [Ca^2+^]_*i*_ at post-stretch between 0 h and 12 h in wild-type (WT) mice. (**A**) Normal conditions. (**B**) In the absence of extracellular Ca^2+^ (Ca^2+^ free balanced salt solution (BSS)). (**C**) In the presence of 10 μM GsMTx4 (GsMTx4 10 μM). (**D**) In the presence of 10 μM RR (Ruthenium Red 10 μM). At 0 h, (N) *vs*. (a), (N) *vs*. (b), and (N) *vs*. (c); P < 0.05, P < 0.01, and P = 0.14 by the Mann–Whitney *U*-test, respectively. At 12 h, (N) *vs*. (a), (N) *vs*. (b), and (N) *vs*. (c); P < 0.01, P < 0.01, and P < 0.01, by the Mann–Whitney *U*-test, respectively. The numbers of analyzed cells are indicated in parenthesis below of the chart. The numbers above error-bar indicate the average of the measured values. Time 0 means 12 hours after horse serum shock (HSS). Statistical analyses were performed using Student’s *t*-test in A and D, Welch’s *t*-test in B, and the Mann–Whitney *u*-test in C. Data are presented as the means ± standard error (SE). * or ^#^*P* < 0.05, **P < 0.01, n.s., not significant.

We investigated the effect of various conditions on the circadian change of average [Ca^2+^]_*i*_ at post-stretch as follows: (N) normal condition; extracted data from Fig. 3, left, at 0 h and 12 h; (a) absence of extracellular Ca^2+^ [Ca^2+^ free balanced salt solution (BSS)], (b) treatment with 10 μM GsMTx4, an inhibitor of Piezo1^7,11^, and (c) treatment with 10 μM Ruthenium Red (RR), an inhibitor of cation channels, including TRPV4^11^ (Figs 4 and 5).

There were no conspicuous differences in the situations of cultured cells such as size and detachment after stretching in conditions (a), (b), and (c) (upper left panels in Fig. 4A–C), compared with condition (N). However, the Fura2 ratio images and the trace charts in conditions (a), (b), and (c) showed that the prominent [Ca^2+^]_*i*_ at post-stretch at 12 h, which was observed in condition (N), had disappeared and the levels remained constant between 0 h and 12 h (upper right and lower panels in Fig. 4A–C).

In conditions (a), (b), and (c), the average [Ca^2+^]_*i*_ at 0 h was attenuated to a similar level among the groups (Fig. 5B–D). The average values of [Ca^2+^]_*i*_ at 0 h were lower than that of condition (N) (Fig. 5). At 12 h, the average [Ca^2+^]_*i*_ was attenuated strongly in condition (a) and was significantly lower than that at 0 h (Fig. 5B). Conditions (b) and (c) also showed prominent attenuation of [Ca^2+^]_*i*_ at 12 h (Fig. 5C and D). The circadian rhythm of the average [Ca^2+^]_*i*_ at post-stretch in condition (N) was abolished in the three treated groups.

## Discussion

We previously confirmed the circadian rhythm of clock genes, *Piezo1* and *TRPV4*, which was regulated by clock genes in MPCUCs^9^. We suggested that one of the contributing factors of nocturia in *Clock*^*Δ19/Δ19*^ mice was loss of circadian expression pattern of mechano-sensors, which send signals of bladder fullness^5,7,8^. The present study demonstrated that the sensation of bladder fullness (SBF), which is caused by [Ca^2+^]_*i*_, might have a circadian rhythm in MPCUCs. In WT mice, SBF becomes sensitive in the active phase and dose not in the sleep phase. Meanwhile, abnormalities in clock genes cause loss of the circadian SBF. Thus, abnormality of the clock genes causes loss of circadian SBF, which induces nocturia^3,9^.

In the Ca^2+^ imaging experiments, a few cells were observed to be removed from the silicon chamber at post-stretch (live images in Figs 1 and 4). These removed cells caused an increased Fura2 ratio change in the cells close to the removed cells. This was possibly because the removed cells stimulate neighboring cells directly, or emitted a substance from that stimulated them indirectly. Epithelial cells, including those in the gut, trachea, and urinary tract, undergo prompt turnover, and are removed from tissues by means of apoptosis and necrosis^12,13^. We hypothesized that intracellular substances were released into extracellular region after cell death induced by removal, which might activate neighboring cells^14^. Indeed, the bladder urothelium under the condition of bacterial cystitis is activated directly by the inflammation, and indirectly by apoptosis-mediated-inflammation, resulting in irritative bladder symptoms^15,16^.

Three criteria in the analysis were applied to exclude the variants and obtain even conditions among the cells. Furthermore, the stretch distance and speed were fixed as 100 μm and 100 μm/s, respectively, in all experiments. Because the change in the field of view of the microscope during the experiments varied too greatly to detect candidate cells for analysis when the stretch distance was longer than 100 μm. A stretch speed faster than 100 μm/s also caused increased variation in circadian change detection by the other stimulations, such as removing cultured cells and unevenly processing in stretch transmission. Using these criteria, the stretch-evoked [Ca^2+^]_*i*_ showed a clear circadian rhythm in WT mice. The time-dependent changes observed in *Clock*^*Δ19/Δ19*^ mice also showed statistical differences. However, this was not the typical circadian rhythm as observed in WT mice (Fig. 3). Furthermore, these rhythms were consistent with the mRNA and protein expression patterns of mechano-sensors in MPCUCs, as reported previously^10^. The mRNA expression rhythm of *Piezo1* in *Clock*^*Δ19/Δ19*^ mice was apparently different from the circadian mRNA expression pattern in WT mice. However, a statistical analysis showed significant differences in time-dependent changes of *Piezo1* mRNA expression pattern in both WT and *Clock*^*Δ19/Δ19*^ mice. Interestingly, this tendency was confirmed by the stretch response results (Fig. 3).

In this study, we applied a single stretch stimulation. However, the response of MPCUCs seemed to vary according to the stretch distance. Miyamoto *et al*. mentioned that a weak stretch, such as 100 μm, was sensed by Piezo1, whereas a stronger stretch over 100 μm was sensed by TRPV4^7^. In addition, this difference might be involved in the sensitivity of their mechano-sensor functions. Piezo1 can sense the stretch stimulation directly^17^, whereas TRPV4 needs to form a molecular complex by binding to the actin cytoskeletal structure and a cell junction to be activated as a mechano-sensor^18^. Thus, it is possible that that circadian rhythm of stretch-evoked [Ca^2+^]_*i*_ associated with a 100 μm-stretch might be driven mainly by the circadian expression of *Piezo1*. In contrast, because the voiding behavior is altered in different conditions, such as cold stress^19^, and osmotic stimuli showed different reactions for stretch in rodents^7^, the circadian rhythm of TRPV4 might contribute to another circadian function in the bladder, such as diurnal change of urine sensation accompanying temperature variation^20^, blood flow to regulate vasodilation^21^, and barrier homeostasis from external stimuli, urine osmotic, or pH change in the dark and light cycle^22,23^.

The way of stretch stimulation may influence the result. Some cells were recovered to the initial level after increasing, which were considered to be normal, however some remained high (Fig. 2). We analyzed the duration of Fura2 ratio increasing to see whether these factors could show circadian rhythm since rhythmic Ca^2+^ dynamics was reported to play an important role that determined not only daily activity rhythm but also the regular circadian clock function in mice^24^. However, we could not see any differences in time-dependent change. For another reason of difference in Fura2 ratio recovery pattern, the normalizing in each cell culture might be insufficient. We believed that cell condition was as close to a certain state as possible, however, there were many factors that were difficult to normalize such as cell overlap and degree of adhesion. These might influence the reaction to the stretch stimulation. By advanced experiment, it may be possible to prove the circadian rhythm of the retaining period in intracellular Ca^2+^ concentration, which determine the duration of urine sensation.

The circadian stretch-evoked [Ca^2+^]_*i*_ in WT mice was abolished in Ca^2+^ free BSS, and 10 μM GsMTx4, and 10 μM RR treated condition (Figs 4 and 5). The average Fura2 ratio change at 0 h and 12 h were lower than those under normal conditions (Fig. 5). These results indicated that the circadian rhythm of stretch-evoked [Ca^2+^]_*i*_ was associated with the function of Pizeo1 and TRPV4. GsMTx4 can inhibit not only Pizeo1, but also various types of stretch-activated-channels (SACs). However, TRPV4 is not inhibited by GsMTx4^7,25^. By contrast, RR is a more non-specific SAC inhibitor that can block both Piezo1 and TRPV4^7,8,26^. If RR was a specific inhibitor of TRPV4, the suggestion that TRPV4 is involved in the regulating the circadian rhythm of SBF would be more reliable. Considering the result showing the abrogation of the circadian rhythm of stretch-evoked [Ca^2+^]_*i*_ after RR administration, SACs other than TRPV4 are more likely to participate in creating the circadian rhythm of SBF. However, these results suggest that the loss of circadian rhythm in SBF is one of the factors that contribute to nocturia, and *Clock*^*Δ19/Δ19*^ mice might sense a constant level of SBF throughout the day. This would explain why the *Clock*^*Δ19/Δ19*^ mice voided with the same frequency in the dark and light and why the circadian change of bladder capacity between dark and light was not observed in *Clock*^*Δ19/Δ19*^ mice^3^.

It is controversial to discuss the phenomenon observed in an *in vitro* experiment as an *in vivo* phenotype. However, the diurnal change in urination pattern is affected by many factors such as functional bladder capacity and bladder smooth muscle contraction^2,27^. Urine production in the kidney is also one of the factors that determine urination behavior^4,28^. Moreover, in addition to activation in local organs, the sensitivity of the central nervous system or the external environment, such as temperature and irregular life style, also promote urination^19,29,30^. Interestingly, the influences of these factors on the circadian clock in local organs often lead to various body symptoms^1,31^. Although SBF signal that is integrated in central nervous system resulting in voiding behavior and the mechanisms between each oscillator are left undetermined, we suggest that Ca^2+^ dynamics associated with gene expression rhythm is one of important contributor to determine SBF. Thus, other factors than the circadian rhythm of SBF, which may also contribute to nocturia, should be identified. Further investigations are needed to reveal the underlying mechanism of the relationship between the circadian clock in the bladder and nocturia.

For another limitation in the present study, the silicon chamber coatings were different between WT and *Clock*^*Δ19/Δ19*^ mice. We used Cell Tak (CORNING, Corning, NY) only in *Clock*^*Δ19/Δ19*^ mice because of the difficulty to maintain adhesion during stretching. This difference might alter the cellular property in stretched reaction. However, we did not compare the differences of stretch-evoked [Ca^2+^]_*i*_ level between WT and *Clock*^*Δ19/Δ19*^ mice directly, then we concluded circadian rhythm of stretch-evoked [Ca^2+^]_*i*_ in the WT and their loss in *Clock*^*Δ19/Δ19*^ mice (Fig. 3). It is not contradictory to state that SBF may have the circadian rhythm associated with the expression rhythm of *Piezo1* and *TRPV4* under the regulation of clock genes.

In conclusion, the circadian SBF is created by a circadian rhythm of [Ca^2+^]_*i*_ through mechano-sensors in MPCUCs, which are regulated by clock genes, are associated with sensitivity in the active phase and insensitivity in the sleep phase. In contrast, the change of circadian rhythm of [Ca^2+^]_*i*_ leads constant urination, which might be one of the factors contributing to nocturia. Thus, remodeling of abnormalities in clock genes, which regulates [Ca^2+^]_*i*_ through mechano-sensors, which might have a great potential as a new target in nocturia.

## Materials and Methods

### Animals

Eight to twelve-week-old male C57BL/6 WT mice and age and sex-matched C57BL/6 *Clock*^*Δ19/Δ19*^ were used in the following experiments. *Clock*^*Δ19/Δ19*^ mice have an A to T mutation in the 5′ splice site of intron 19 of the *Clock* gene, and an in-frame deletion of entire exon 19 (*Clock*^*Δ19/Δ19*^), which results in loss of normal transcriptional activity and show a nocturia phenotype^3^. All experiments were performed using these mice. All procedures were conducted in accordance with the “Guiding Principles in the Care and Use of Animals in the Field of Physiologic Sciences” published by the Physiologic Society of Japan. In addition, all experimental protocols were approved by the Animal Care Committee of the University of Yamanashi (Chuo, Yamanashi, Japan).

### Preparation of MPCUCs

Urothelial cells were cultivated on elastic silicone chambers (STB-CH-04; STREX, Osaka, Japan) using previously described methods with slight modifications (Supplementary Figure 1A)^7,8^. Coating with 50 µg/mL Fibronectin (WAKO, Osaka, Japan) was performed in WT mice. In case of *Clock*^*Δ19/Δ19*^ mice, 50 µg/mL Fibronectin and CELL-TAK (CORNING) was used.

To reset and synchronize the gene expression rhythms in each cell, 50% heat inactivated horse serum (Gibco^TM^, Thermo Fisher Scientific, Waltham, MA) was added for 2 h (Horse Serum Shock; HSS). The cells were then maintained in Dulbecco’s modified Eagle medium (DMEM) without Phenol Red (WAKO) with 1% Penicillin/Streptomycin (P/S; Gibco^TM^). Experiments were performed 12 h after HSS. The time of the first experiment was defined as 0 h (12 hours after HSS). For the *in vitro* time course, the light period was set from 0 h to 12 h, and the dark period was set from 12 h to 20 h, according to gene expression rhythm in the mouse bladder mucosa, in which peak time was observed at zeitgeber time 12^9,10^.

### Mechanical stretch experiment

The mechanical stretch experiment was conducted as previously described using elastic silicon chambers and an extension device (STREX) (Supplementary Figure. 1)^7,8^. After the chambers were attached to the extension device and detection reagents or solutions were added, they were left for 15 min to avoid stimulation by the moving or adding of the reagents or solutions. All experiments were done at room temperature and the cell extension system was fixed at a stretch distance of 100 μm, and a stretch speed of 100 μm/s in all experiments.

### Measurement of intracellular Ca^2+^ influx ([Ca^2+^]_*i*_)

MPCUCs were loaded with the fluorescent Ca^2+^ indicator Fura-2 AM (10 μM; Life Technologies, Carlsbad, CA) with 0.04% Pluronic F-127 (Sigma-Aldrich, St. Louis, MO) for 60 min at room temperature. The cells were then washed with balanced salt solution (BSS). 10 μM GsMTx4 (Peptide Institute, INC., Osaka, Japan) and 10 μM RR (WAKO) were applied directly into the chamber. Measurement of [Ca^2+^]_*i*_ was performed for 5 min at pre- and post-stretch stimulation, using previously described methods with slight modifications^7,8^. The ratio-changes were calculated by subtracting basal values from the peak values. The analysis conditions were required to satisfy the following three criteria. 1; The basal values of the Fura2 ratio must be less than 1.5. 2; The cell-cultures must be larger than the field view of the microscope. 3; When the cells were not fixed on the silicon chamber bottom at post-stretch, the cells around them were excluded from the analysis.

### Statistical analyses

Experimental values were expressed as means ± standard error (SE). The significances of the differences between two groups were analyzed using Student’s *t*-test, Welch’s *t*-test, or Mann–Whitney’s *U*-test, depending on the distribution of the samples. A one-way analysis of variation (ANOVA) was used to compare differences among the time points in each group. A *P* value of less than 0.05 was considered significant.

## Electronic supplementary material

Supplementary figure
