# Supplementary material for: The oscillation of intracellular Ca2+ influx associated with the circadian expression of Piezo1 and TRPV4 in the bladder urothelium
Source: Sci Rep. 2018 Apr 9;8:5699. doi: 10.1038/s41598-018-23115-w (PMC5890282; doi:10.1038/s41598-018-23115-w)
Supplement: Supplementary file 1 — Supplementary figure [file 41598_2018_23115_MOESM1_ESM.pdf]

Title;

The oscillation of intracellular Ca^{2+} influx associated with the circadian expression of *Piezo1* and *TRPV4* in the bladder urothelium.

Tatsuya Ihara[1], Takahiko Mitsui[1], Yuki Nakamura[2], Mie Kanda[1], Sachiko Tsuchiya[1], Satoru Kira[1], Hiroshi Nakagomi[1], Norifumi Sawada[1], Manabu Kamiyama[1], Yuri Hirayama[3], Eiji Shigetomi[3], Youichi Shinozaki[3], Mitsuharu Yoshiyama[1], Atsuhito Nakao[2], Masayuki Takeda[1*] and Schuichi Koizumi[3*]

[1]Department of Urology, Interdisciplinary Graduate School of Medicine, University of Yamanashi, Chuo, Yamanashi, Japan

[2]Department of Immunology, Interdisciplinary Graduate School of Medicine, University of Yamanashi, Chuo, Yamanashi, Japan

[3]Department of Neuropharmacology, Interdisciplinary Graduate School of Medicine, University of Yamanashi, Chuo, Yamanashi, Japan

*Correspondence author:

Schuichi Koizumi

1110 Shimokato, Chuo, Yamanashi, 409-3898 Japan

Tel: +81-55-273-9503

Fax: +81-55-273-6739

E-mail: skoizumi@yamanashi.ac.jp

Masayuki Takeda

1110 Shimokato, Chuo, Yamanashi, 409-3898 Japan

Tel: +81-55-273-9643

Fax: +81-55-273-9659

E-mail: matakeda@yamanashi.ac.jp

Supplementary Figure 1

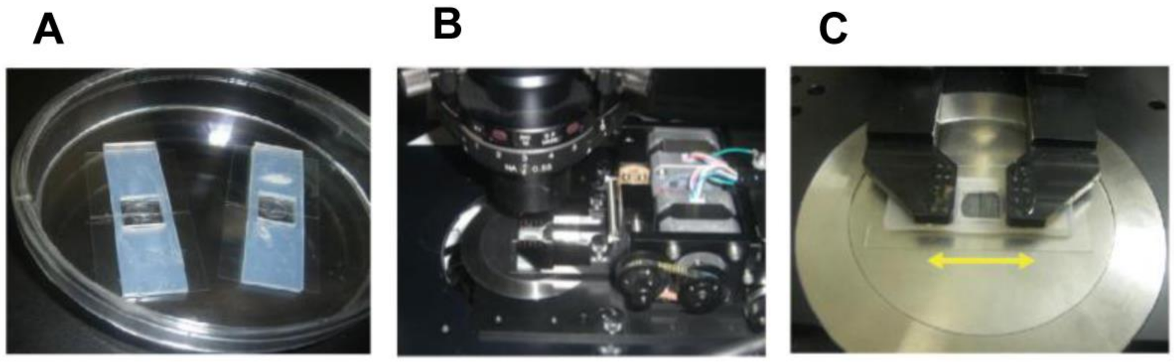

Supplementary Figure 1. ***In vitro*** **cell stretch system.**

A, Elastic silicone chambers. B and C, The silicone chamber was set in the 2 arms of the extension device on the microscope stage. The arrow in C indicates the direction of extension.
